# Risk scores and coronary artery disease in patients with suspected acute coronary syndrome and intermediate cardiac troponin concentrations

**DOI:** 10.1136/openhrt-2024-002755

**Published:** 2024-08-01

**Authors:** Daniel Perez-Vicencio, Alexander J F Thurston, Dimitrios Doudesis, Rachel O'Brien, Amy Ferry, Takeshi Fujisawa, Michelle Claire Williams, Alasdair J Gray, Nicholas L Mills, Kuan Ken Lee

**Affiliations:** 1 Centre for Cardiovascular Science, The University of Edinburgh, Edinburgh, UK; 2 Usher Institute, The University of Edinburgh, Edinburgh, UK; 3 Emergency Medicine Research Group, Department of Emergency Medicine, Royal Infirmary of Edinburgh, Edinburgh, UK

**Keywords:** Acute Coronary Syndrome, Coronary Artery Disease, Computed Tomography Angiography, Chest Pain, Risk Factors

## Abstract

**Background:**

Guidelines recommend the use of risk scores to select patients for further investigation after myocardial infarction has been ruled out but their utility to identify those with coronary artery disease is uncertain.

**Methods:**

In a prospective cohort study, patients with intermediate high-sensitivity cardiac troponin I concentrations (5 ng/L to sex-specific 99th percentile) in whom myocardial infarction was ruled out were enrolled and underwent coronary CT angiography (CCTA) after hospital discharge. History, ECG, Age, Risk factors, Troponin (HEART), Emergency Department Assessment of Chest Pain Score (EDACS), Global Registry of Acute Coronary Event (GRACE), Thrombolysis In Myocardial Infarction (TIMI), Systematic COronary Risk Evaluation 2 and Pooled Cohort Equation risk scores were calculated and the odds ratio (OR) and diagnostic performance for obstructive coronary artery disease were determined using established thresholds.

**Results:**

Of 167 patients enrolled (64±12 years, 28% female), 29.9% (50/167) had obstructive coronary artery disease. The odds of having obstructive disease were increased for all scores with the lowest and highest increase observed for an EDACS score ≥16 (OR 2.2 (1.1–4.6)) and a TIMI risk score ≥1 (OR 12.9 (3.0–56.0)), respectively. The positive predictive value (PPV) was low for all scores but was highest for a GRACE score >88 identifying 39% as high risk with a PPV of 41.9% (30.4–54.2%). The negative predictive value (NPV) varied from 77.3% to 95.2% but was highest for a TIMI score of 0 identifying 26% as low risk with an NPV of 95.2% (87.2–100%).

**Conclusions:**

In patients with intermediate cardiac troponin concentrations in whom myocardial infarction has been excluded, clinical risk scores can help identify patients with and without coronary artery disease, although the performance of established risk thresholds is suboptimal for utilisation in clinical practice.

**Trial registration number:**

NCT04549805.https://clinicaltrials.gov/study/NCT04549805

**WHAT IS ALREADY KNOWN ON THIS TOPIC**

- Clinical risk scores have been recommended in the assessment of patients with intermediate troponin concentrations.

**WHAT THIS STUDY ADDS**

- Clinical risk scores improve the odds of identifying coronary artery disease in patients with intermediate troponin concentrations.
- The positive and negative predictive values of established risk score thresholds are suboptimal for the identification of coronary artery disease.

**HOW THIS STUDY MIGHT AFFECT RESEARCH, PRACTICE OR POLICY**

- Development of novel methods specifically for this purpose will be required to risk stratify patients with intermediate troponin concentrations for further investigation most effectively.

## Introduction

 Current strategies to assess patients with the suspected acute coronary syndrome in the emergency department primarily use high-sensitivity cardiac troponin testing and the ECG to rule in and rule out myocardial infarction.^1 2^ Early rule-out pathways have been developed to identify patients with low cardiac troponin concentrations who can be safely discharged from the emergency department without further testing and those with elevated concentrations who require admission for further assessment.^3^,^8^ However, one in four patients have intermediate cardiac troponin concentrations and while an acute coronary syndrome can be ruled out or ruled in with a second troponin measurement, they remain at higher risk of future adverse cardiac events.^59^,^11^ Similarly, serial testing pathways with cardiac troponin measurement at 0/1 and 0/2 hours stratify patients into three groups, including an observe zone group with intermediate concentrations.^12 13^ Guidelines recommend further investigation should be considered in these patients but the optimal approach to select these patients for cardiac imaging is unknown.^2 14^

Clinical risk scores are widely used in the emergency department to risk stratify patients with suspected acute coronary syndrome.^15^,^18^ However, the utility of these risk scores is uncertain, particularly following the widespread adoption of high-sensitivity cardiac troponin as a risk stratification tool.^19^ Derived from historical cohorts, risk scores may lack external validity when applied to contemporary practice,^20^ and some include elements of the history or clinician gestalt that may be subjective or vulnerable to bias. Nevertheless, recent guidelines continue to recommend the use of clinical risk scores to select patients with intermediate cardiac troponin concentrations for further testing.^1 2^

In a secondary analysis of the PRECISE-CTCA study,^21^ we evaluate the performance of clinical risk scores to identify coronary artery disease in patients with intermediate cardiac troponin concentrations in whom myocardial infarction has been ruled out.

## Methods

### Study design and population

PRECISE-CTCA (Troponin to Risk Stratify Patients with Acute Chest Pain for CT Coronary Angiography) was a prospective cohort study conducted at the Royal Infirmary of Edinburgh, UK, between 4 December 2018 and 6 October 2020 (ClinicalTrials.gov, number NCT04549805).^21^ Patients >30 years old presenting to the Emergency Department with suspected acute coronary syndrome, in whom myocardial infarction had been ruled out and peak cardiac troponin concentrations within the normal reference range were eligible for this study.^21^ Patients who were unable to undergo CCTA due to severe renal failure (estimated glomerular filtration rate <30 mL/min/1.73 m^2^) or a major allergy to iodinated contrast media, a clear alternative diagnosis, a requirement for in-patient investigation, a CCTA or invasive coronary angiogram within the past 1 year, pregnancy or breast feeding and an inability to give informed consent were excluded.

Only patients with intermediate cardiac troponin concentrations (between 5 ng/L and the sex-specific 99th percentile) were included in this secondary analysis. Cardiac troponin was measured using the ARCHITECT_STAT_ high-sensitivity cardiac troponin I assay (Abbott Laboratories, Abbott Park, Illinois, USA). This assay has a limit of detection of 1.2 ng/L and an inter-assay coefficient of variation of <10% at 4.7 ng/L, with a sex-specific upper reference limit or 99th percentile of 16 ng/L in females and 34 ng/L in males.^22^ Presenting symptoms, cardiovascular risk factors, medical history, physiological measurements, clinical biochemistry and haematologic, and prescribed medications were recorded from participants at enrolment and from their electronic medical records. According to current national and international recommendations, symptoms of angina were classified as typical, atypical or non-anginal chest pain using the Diamond and Forrester questions.^23^

### Coronary CT angiography

All participants underwent CCTA as an outpatient as soon as possible after their initial hospital attendance. CCTA was performed using a 128-detector row scanner (Biograph mCT, Siemens Healthcare, Germany) with iodine-based contrast media, as per Society of Cardiovascular Computed Tomography (SCCT) guidelines.^24^ All CCTA images were analysed by trained observers who performed a per-segment analysis using a 15-segment model to assess coronary artery stenoses. Luminal cross-sectional area stenoses were classified as normal (<10%), mild non-obstructive (10–49%), moderate non-obstructive (50–70%) or obstructive (>70% in ≥1 major epicardial artery or >50% in the left main stem). Patients were classified according to the most significant stenosis identified on CCTA, irrespective of whether the vessel had been stented. Coronary stenoses that were bypassed by a vascular graft were not considered in the classification.

### Clinical risk scores

**Figure 1:**
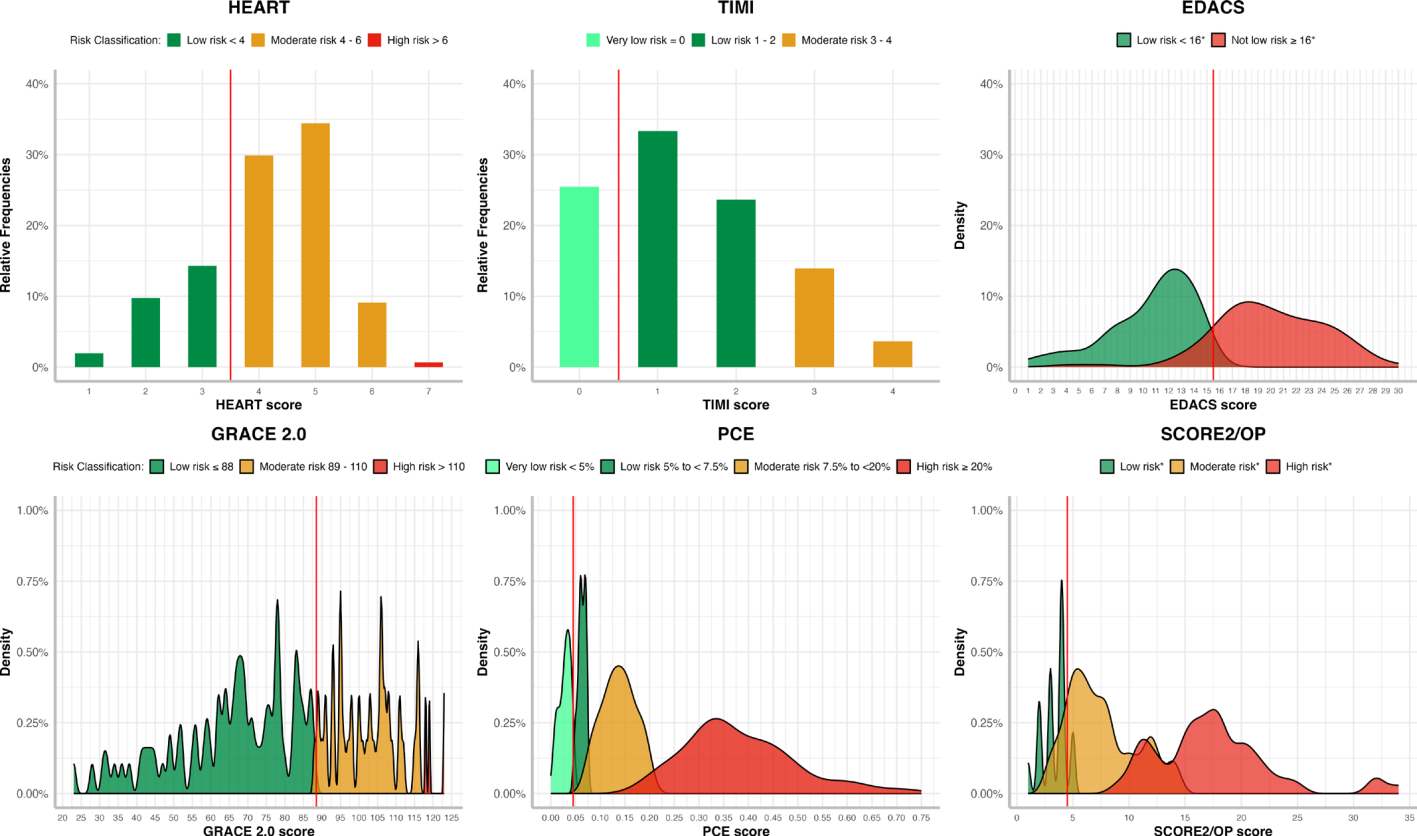
Distribution of risk scores in patients with suspected acute coronary syndrome and intermediate cardiac troponin concentrations stratified using established risk score thresholds as low, moderate or high risk. *When EDACS and SCORE2/OP are applied, further criteria are recommended (online supplemental text 1).^8^ EDACS, Emergency Department Assessment of Chest Pain Score; GRACE 2.0, Global Registry of Acute Coronary Events V.2.0; HEART, History, ECG, Age, Risk factors, Troponin; PCE, Pooled Cohort Equation; SCORE2/OP, Systematic COronary Risk Evaluation 2/Older Population; TIMI, Thrombolysis In Myocardial Infarction.

We calculated the History, ECG, Age, Risk factors, Troponin (HEART), Emergency Department Assessment of Chest Pain Score (EDACS), Global Registry of Acute Coronary Events V2.0 (GRACE 2.0), Thrombolysis In Myocardial Infarction (TIMI), Pooled Cohort Equation (PCE) and Systematic COronary Risk Evaluation 2 (SCORE2) risk scores in all patients and used established thresholds for each to stratify patients as low or high risk (online supplemental text 1 and figure 1).^1725^,^31^ The HEART score assesses the risk of major adverse cardiac events at 6 weeks in patients presenting with chest pain to the emergency department using a threshold of <4 to identify those who are low-risk.^25^ The EDACS score assesses the risk of major adverse cardiac events at 30 days in patients presenting with chest pain to the emergency department using a threshold of <16 to identify those who are low-risk.^17^ The GRACE 2.0 score assesses the risk of death or recurrent myocardial infarction at 6 months in patients with acute coronary syndromes identifying patients with a score >88 at increased risk.^17^ The TIMI score assesses the risk of death, re-infarction or ischaemic events at 14 days in patients with acute coronary syndrome with a score of ≥1 associated with an increased risk.^27^ We also included two risk scores for the prediction of atherosclerotic risk, although they have not been recommended in the assessment of acute chest pain. The PCE predicts the risk of a first atherosclerotic cardiovascular disease event at 10 years with the score associated with a <5% selected as low risk.^28 29^ SCORE2 and SCORE2-OP estimate the risk of fatal cardiovascular disease at 10 years in adults aged 40–69 and 70 years or older, respectively. The SCORE2 and SCORE2-OP risk scores are used together in this analysis and were calibrated for low-risk regions with the score associated with a <5% selected as low risk^30 31^ (online supplemental text 1).

### Statistical analysis

Baseline characteristics were presented as mean±SD or median (IQR) for continuous variables and as count (%) for categorical variables. The Welch two-sample t-test test and one-way analysis of variance were used to compare continuous variables, while Fisher’s exact test was used to compare categorical variables. Multiple imputation by chained equation or Markov chain Monte Carlo method was performed to account for missing variables.^32^ We multiple-imputed all missing values in the variables required to calculate clinical risk scores except for cardiac troponin concentrations (online supplemental figure 2). We evaluated the association between the clinical risk scores and the presence of any coronary artery disease and obstructive coronary artery disease separately using binomial logistic regression modelling by obtaining the exponential of the logistic regression coefficient. We calculated the diagnostic performance for each clinical risk score with 95% CIs of the sensitivity, specificity, negative predictive value (NPV) and positive predictive value (PPV) based on the rule-in/rule-out thresholds. Overall diagnostic accuracy was evaluated by receiver operating characteristic (ROC) curve analysis, with pairwise comparisons of the area under the curve for each risk score with DeLong’s test, for which a Bonferroni-corrected α of 0.05/15=0.0033 was considered significant. All calculations were performed for any coronary artery disease and obstructive disease separately. We subsequently performed a sensitivity analysis restricted to patients who were not previously known to have coronary artery disease (online supplemental table 1). All data analyses were conducted in R (V.4.3.0, R Foundation for Statistical Computing).

## Results

### Study population

In this secondary analysis, 167 patients (64±12 years, 28% female) were included with intermediate cardiac troponin I concentrations and a median maximal concentration of 8 ng/L (IQR 6–12 ng/L). Of these patients, 120 (72%) had coronary artery disease and 50 (30%) had obstructive coronary artery disease on CCTA (table 1).

**Table 1:** Baseline characteristics of patients with intermediate cardiac troponin concentrations stratified by findings on coronary CT angiography

	All participants(n=167)	No coronary artery disease(n=47)	Any coronary artery disease(n=120)	P value*
Female sex	46 (28%)	19 (40%)	27 (23%)	0.033
Age, years	64 (12)	56 (13)	67 (10)	<0.001
Presenting symptom				
Chest pain	143 (86%)	37 (79%)	106 (88%)	0.14
Anginal symptoms	76 (46%)	21 (45%)	55 (46%)	0.99
Cardiovascular risk factor				
BMI, kg/m^2^	29.3 (5.8)	29.6 (5.2)	29.2 (6.0)	0.73
Current or previous smoker	89 (53%)	18 (38%)	71 (59%)	0.017
Hypertension	79 (47%)	11 (23%)	68 (57%)	<0.001
Diabetes	30 (18%)	3 (6.4%)	27 (23%)	0.014
Hyperlipidaemia	33 (20%)	10 (22%)	23 (19%)	0.83
Family history	64 (38%)	17 (36%)	47 (39%)	0.86
Chronic kidney disease	19 (11%)	2 (4.3%)	17 (14%)	0.010
Medical history				
Angina	34 (20%)	2 (4.3%)	32 (27%)	0.001
Myocardial infarction	40 (24%)	3 (6.4%)	37 (31%)	<0.001
Stroke	13 (7.8%)	2 (4.3%)	11 (9.2%)	0.36
Peripheral vascular disease	6 (3.6%)	0 (0%)	6 (5.0%)	0.19
Previous revascularisation				
PCI	40 (24%)	3 (6.4%)	37 (31%)	<0.001
CABG	10 (6.0%)	0 (0%)	10 (8.3%)	0.064
Physiology and investigations				
Ischaemia on ECG	8 (4.8%)	3 (6.4%)	5 (4.2%)	0.69
T-wave inversion	24 (15%)	5 (11%)	19 (16%)	0.62
Heart rate, bmp	76 (18)	77 (20)	76 (18)	0.87
Systolic BP, mm Hg	151 (27)	152 (22)	151 (29)	0.90
Haemoglobin, g/L	143 (15)	143 (16)	143 (14)	0.95
Creatine	82 (19)	79 (19)	83 (19)	0.25
eGFR, mL/min/1.73 m2	82 (18)	87 (18)	80 (17)	0.022
Total cholesterol, mmol/L	4.82 (1.18)	5.18 (0.95)	4.67 (1.23)	0.005
LDL cholesterol, mmol/L	2.96 (1.18)	3.30 (0.94)	2.84 (1.24)	0.011
Peak cardiac troponin I, ng/L	8 (6–12)	8 (7–13)	8 (6–12)	0.70
Clinical risk scores				
HEART	4 (3–5)	3 (2–4.5)	5 (4–5)	<0.001
EDACS	17 (12–21)	14 (10.8–17)	18 (13–21.3)	<0.001
GRACE	78 (64–96)	62 (45–73)	86 (69–102)	<0.001
TIMI	1 (0–2)	0 (0–1)	2 (1–2)	<0.001
PCE	0.19 (0.08–0.36)	0.09 (0.04–0.16)	0.26 (0.13–0.38)	<0.001
SCORE2/OP	10.5 (5, 16)	5 (4, 9)	12.5 (7, 18)	<0.001

Values are median (interquartile rangeIQR), n (%) or mean±SD.SD.

* Pearson’s Chi-squaredχ2 test; Wilcoxon rank sum test; Fisher’s exact test.

2.0, Global Registry of Acute Coronary Events V.2.0HEARTBMI, body mass index; BP, blood pressure; bpm, beats per minute; CABG, coronary artery bypass grafting; CAD, coronary artery disease; ECG, electrocardiogram; EDACS, Emergency Department Assessment of Chest Pain Score; eGFR, estimated glomerular filtration rate; GRACE 2.0Global Registry of Acute Coronary Events V.2.0HEARTHistory, ECG, Age, Risk factors, TroponinLDLlow-density lipoproteinPCEPooled Cohort EquationPCIpercutaneous coronary interventionSCORE2/OPSystematic COronary Risk Evaluation 2/Older PopulationTIMIThrombolysis In Myocardial Infarction

Patients with coronary artery disease were older than those without (67±10 years vs 56±13 years, respectively; p<0.001) and more likely to be current or previous smokers (59% (71/120) vs 38% (18/47); p=0.017). Patients with coronary artery disease were also more likely to have hypertension (57% (68/120) vs 23% (11/47); p=0.017), diabetes mellitus (23% (27/120) vs 6.4% (3/47); p=0.014) and chronic kidney disease (14% (17/120) vs 4.3% (2/47); p=0.010) compared with those without (table 1). Similarly, patients with coronary artery disease were also more likely to have symptoms of typical angina (27% (32/120) vs 4.3% (2/47); p=0.001) and to have both previous myocardial infarction (31% (37/120) vs 6.4% (3/47); p<0.001) and percutaneous coronary intervention (31% (37/120) vs 6.4% (3/47); p<0.001) than those without (table 1). Similar findings were observed when stratified according to the presence or absence of obstructive coronary artery disease (online supplemental table 2).

### Distribution of clinical risk scores

The median scores for patients with the obstructive disease were significantly higher than those without coronary artery disease for all risk scores; HEART (5 (IQR 4–5) vs 3 (2–4.5); p<0.001), EDACS (18 (14–24) vs 14 (10.8–17); p=0.002), GRACE (88 (77–108) vs 62 (45–73); p<0.001), TIMI (2 (1–3) vs 0 (0–1); p<0.001), PCE (0.32 (0.16–0.45) vs 0.09 (0.04–9); p<0.001) and SCORE2 (15 (8.5–18.8) vs 5 (4–9); p<0.001), respectively (figure 1, online supplemental table 2). Similarly, patients with obstructive coronary artery disease had higher median scores than those with non-obstructive disease (online supplemental table 2).

### Diagnostic performance of clinical risk scores

**Figure 2:**
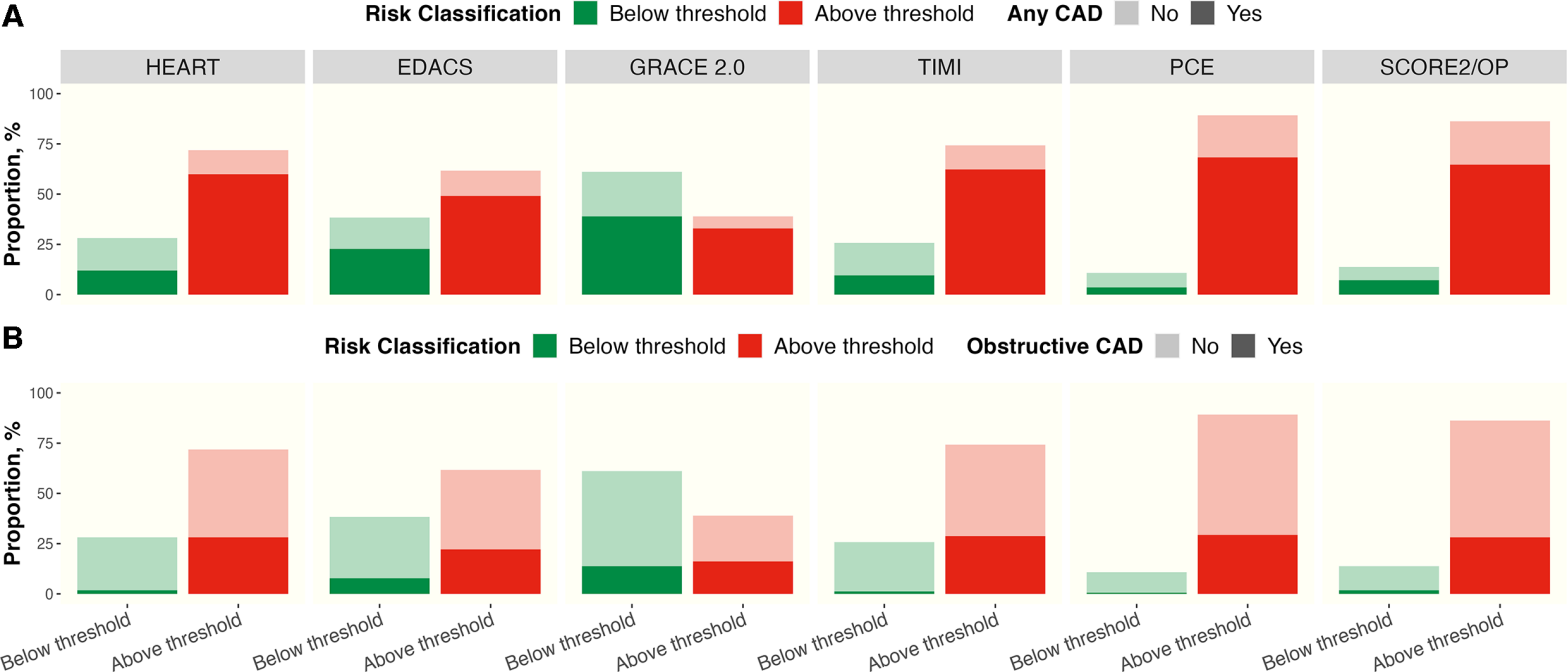
Proportion of patients with suspected acute coronary syndrome and intermediate cardiac troponin concentrations found to have any coronary artery disease (panel A) or obstructive disease (panel B) on CCTA below or above established low-risk thresholds for each risk score. *When EDACS and SCORE2/OP are applied, further criteria are recommended (online supplemental text 1). CAD, coronary artery disease; CCTA, coronary computer tomography angiography; EDACS, Emergency Department Assessment of Chest Pain Score; GRACE 2.0, Global Registry of Acute Coronary Events V.2.0; HEART, History, ECG, Age, Risk factors, Troponin; PCE, Pooled Cohort Equation; SCORE2/OP, Systematic COronary Risk Evaluation 2/Older Population; TIMI, Thrombolysis In Myocardial Infarction.

**Figure 3:**
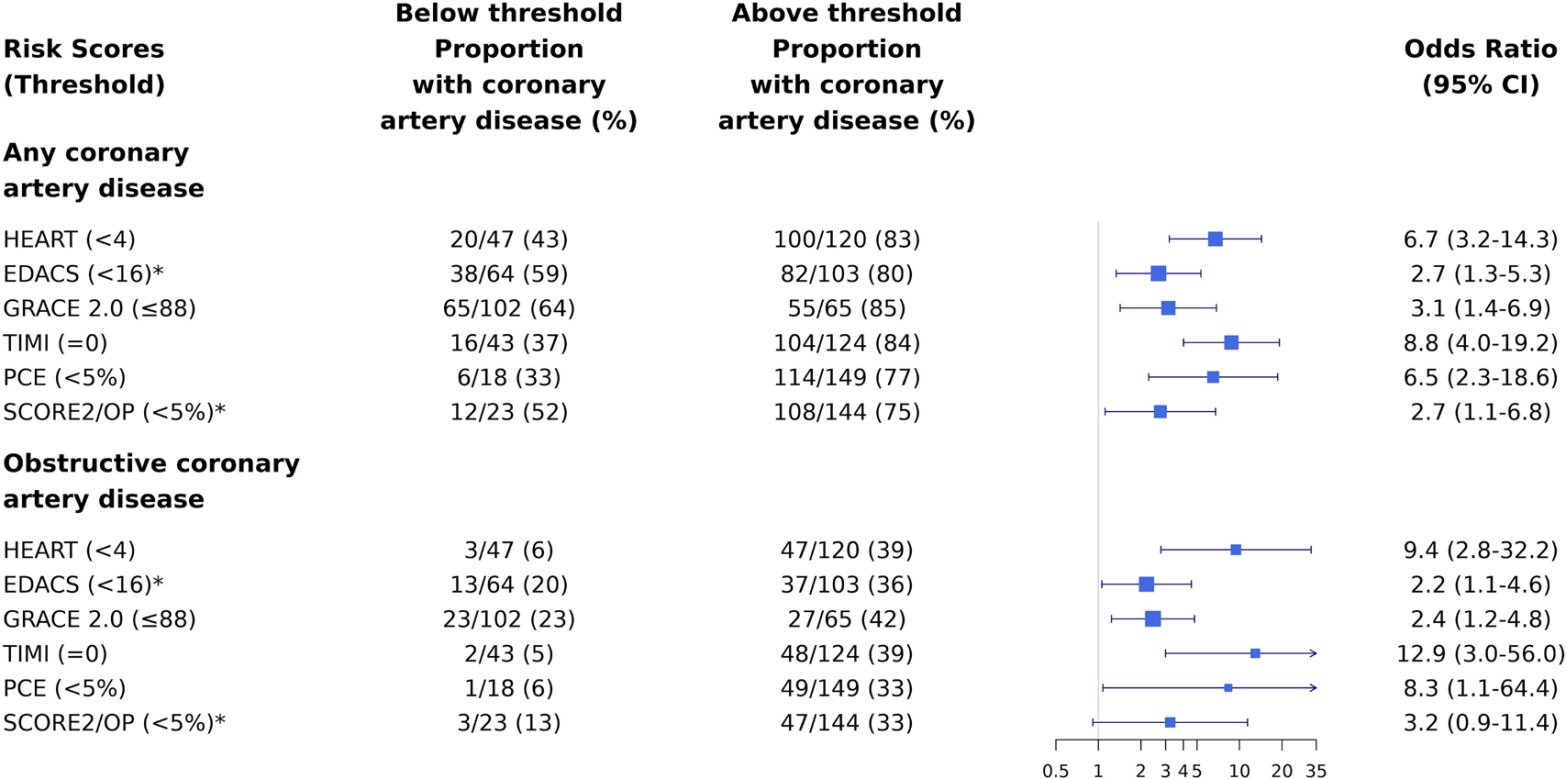
OR of having any or obstructive coronary artery disease on CCTA in patients with suspected acute coronary syndrome and intermediate cardiac troponin concentrations stratified by risk scores. ORs for any coronary artery disease and obstructive disease on CCTA in patients with intermediate cardiac troponin concentrations comparing those with scores above and below established risk thresholds. CCTA, coronary computer tomography angiography; EDACS, Emergency Department Assessment of Chest Pain Score; GRACE 2.0, Global Registry of Acute Coronary Events V.2.0; HEART, History, ECG, Age, Risk factors, Troponin; PCE, Pooled Cohort Equation; SCORE2/OP, Systematic COronary Risk Evaluation 2/Older Population; TIMI, Thrombolysis In Myocardial Infarction.

Patients with risk scores above the established risk threshold were more likely to have coronary artery disease than those below the risk threshold (figure 2). The OR of having any coronary artery disease or obstructive disease was increased for all scores comparing those with increased scores to those with scores below the risk threshold. The OR for obstructive coronary artery disease varied with the lowest increase observed for an EDACS score ≥16 (OR 2.2 (1.1–4.6)) and the highest increase for a TIMI risk score ≥1 (OR 12.9 (3.0–56.0)). Similarly, the OR for any coronary artery disease varied with the lowest increase observed for an EDACS score ≥16 (OR 2.7 (1.3–5.3)) and the highest increase for a TIMI risk score ≥1 (OR 8.8 (4.0–19.2)), respectively (figure 3).

**Figure 4:**
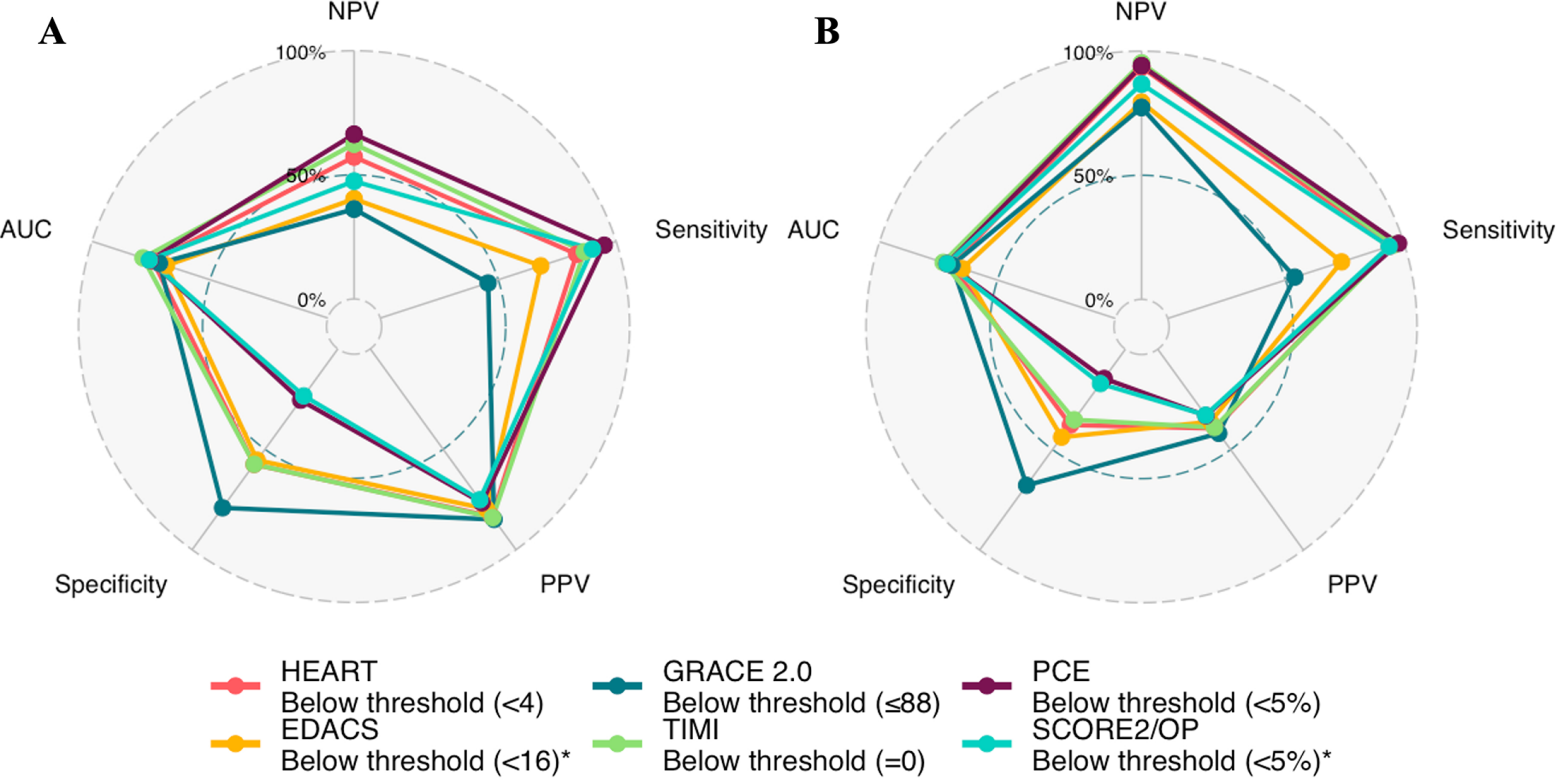
Radar plot comparing diagnostic performance of risk scores for any coronary artery disease (panel A) and obstructive disease (panel B) in patients with suspected acute coronary syndrome and intermediate cardiac troponin concentrations. AUC, area under the curve; EDACS, Emergency Department Assessment of Chest Pain Score; GRACE 2.0, Global Registry of Acute Coronary Events V.2.0; HEART, History, ECG, Age, Risk factors, Troponin; NPV, negative predictive value; PCE, Pooled Cohort Equation; PPV, positive predictive value; SCORE2/OP, Systematic COronary Risk Evaluation 2/Older Population; TIMI, Thrombolysis In Myocardial Infarction.

The PPV was low for all scores, and the NPV varied widely. Across all clinical risk scores, a GRACE 2.0 score of >88 had the highest PPV for obstructive coronary artery disease, identifying 39% as high risk with a PPV of 41.9% (30.4–54.2% CI). The NPV varied from 77.3% to 95.2% but was highest for a TIMI score of 0, identifying 26% as low risk with an NPV of 95.2% (87.2–100%) (figure 4, online supplemental table 3). Similar findings were observed when considering any coronary artery disease (online supplemental table 4).

### High-sensitivity troponin I versus clinical risk scores

**Figure 5:**
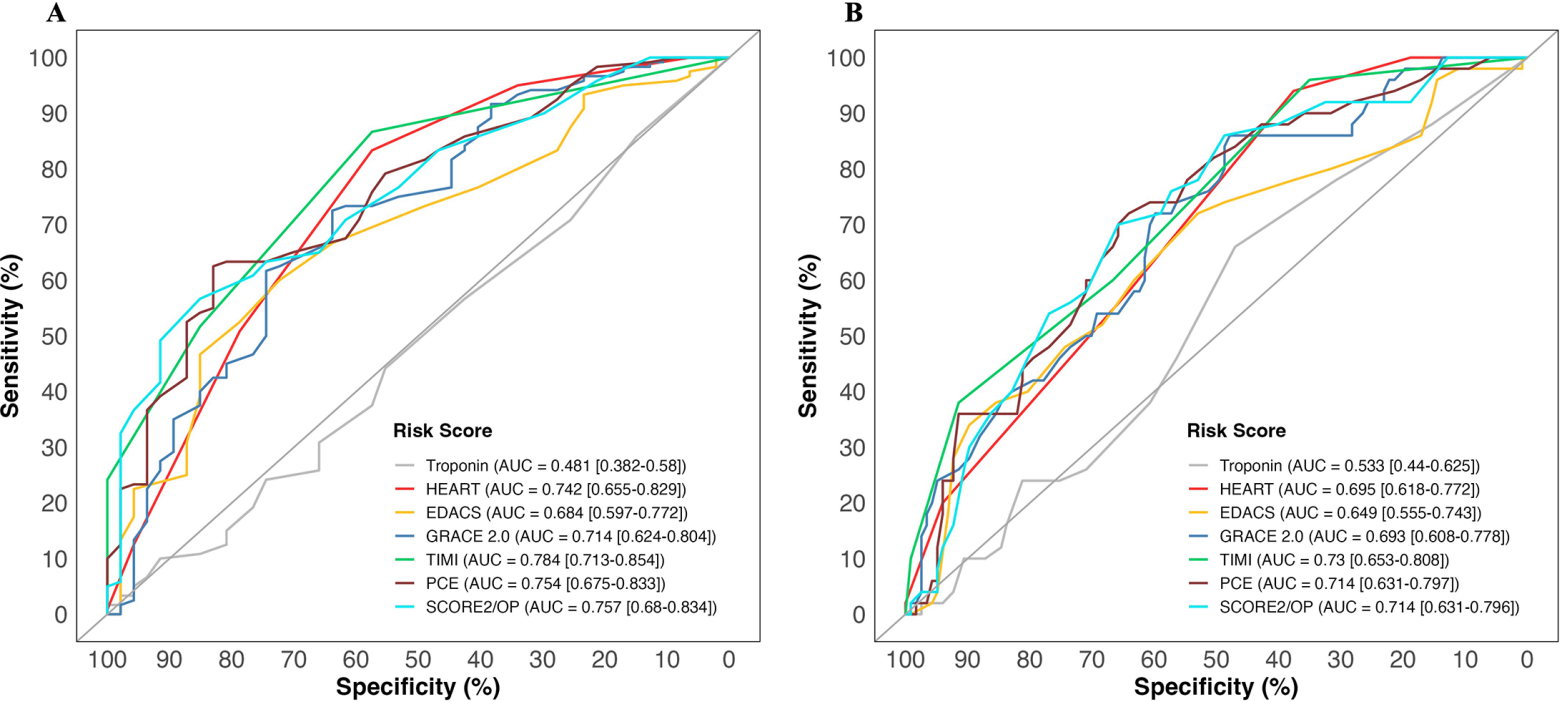
Discrimination of cardiac troponin and clinical risk scores for any coronary artery disease (panel A) and obstructive disease (panel B) in patients with suspected acute coronary syndrome and intermediate cardiac troponin concentrations. Receiver-operating characteristic curves illustrating discrimination of cardiac troponin and clinical risk scores for coronary artery disease (panel A) and obstructive disease (panel B) in patients with suspected acute coronary syndrome and intermediate cardiac troponin concentrations. AUC, area under receiver operator curve; EDACS, Emergency Department Assessment of Chest Pain Score; GRACE 2.0, Global Registry of Acute Coronary Events V.2.0; HEART, History, ECG, Age, Risk factors, Troponin; PCE, Pooled Cohort Equation; SCORE2/OP, Systematic COronary Risk Evaluation 2/Older Population; TIMI, Thrombolysis In Myocardial Infarction.

All clinical risk scores had a higher discriminatory performance than intermediate-range high-sensitivity cardiac troponin alone (area under receiver operator curve (AUC) 0.481 (0.383–0.580 CI) and 0.533 (0.440–0.625) for any coronary artery disease and obstructive disease, respectively). The TIMI risk score had the highest discrimination for coronary artery disease and obstructive disease (0.784 (0.713–0.854) and 0.730 (0.653–0.808), respectively). The lowest performing clinical risk score was EDACS for both coronary artery disease and obstructive disease (0.684 (0.597–0.772) and 0.649 (0.555–0.743), respectively) (figure 5). There were no significant differences among the risk scores themselves (all pairwise p values >0.05/15) (online supplemental figure 3).

### Patients without previous diagnosis of coronary artery disease

In a sensitivity analysis restricted to the 103 (62%) patients not previously known to have coronary artery disease, the discrimination for coronary artery disease and obstructive disease of all risk scores was higher than cardiac troponin alone (cardiac troponin, AUC=0.472 (0.383–0.580) and 0.584 (0.440–0.625), respectively). Discrimination was greatest for SCORE2 for the outcome of any coronary artery disease (0.753 (0.659–0.846) and for the PCE for the outcome of obstructive disease (0.747 (0.639–0.856)). EDACS again had the lowest discrimination for coronary artery disease and obstructive disease (0.658 (0.551–0.764) and 0.641 (0.488–0.795), respectively) (online supplemental figure 4 and online supplemental tables 5 and 6).

## Discussion

In this study, we evaluated the performance of six established clinical risk scores to determine whether they could help identify patients with intermediate troponin levels who are more likely to have coronary artery disease after myocardial infarction has been ruled out in the emergency department. We found that all risk scores improve the odds of identifying patients with coronary artery disease on CCTA. Using the existing risk threshold for each score, the PPV is low for all scores, with the highest for the GRACE 2.0 score, where 4 in 10 high-risk patients have obstructive disease. The NPV was also low, with the best performing score being TIMI, which correctly identifies 19 of 20 patients as not having obstructive disease.

Patients with the suspected acute coronary syndrome are at risk of future myocardial infarction or cardiac death even after myocardial infarction has been ruled out; therefore, clinical guidelines recommend further non-invasive investigations to identify potential underlying coronary artery disease.^5 14^ CCTA has been suggested as the non-invasive investigation modality of choice due to its ability to accurately assess coronary artery plaque burden and characteristics to guide the use of secondary preventative therapies such as antiplatelets and statins to modify their risk of future major adverse cardiovascular outcomes.^233^,^35^ However, given resource constraints and the large volume of patients presenting with suspected acute coronary syndrome, it would be valuable to develop strategies to select patients with a higher pre-test probability of coronary artery disease to avoid unnecessary CCTA. In our previous analysis, we demonstrated that high-sensitivity cardiac troponin I can help identify patients with a higher prevalence of coronary artery disease for further testing after myocardial infarction has been ruled out. Those with intermediate cardiac troponin concentrations had three times higher odds of having coronary artery disease compared with those with very low troponin concentrations. However, this remains a substantial group of patients, comprising approximately one in four of all patients with suspected acute coronary syndrome in whom cardiac troponin testing alone does not further discriminate those who are likely to have coronary artery disease. Strategies to refine risk in this group of patients could therefore help target further investigations more judiciously.

Multiple risk scores have been developed and validated for the initial triage of patients with suspected acute coronary syndrome and for the risk stratification of apparently healthy individuals.^517 21 25^,^31^ These risk scores are recommended by clinical guidelines to guide early referral for specialist investigation such as invasive coronary angiography or the initiation of preventative medications.^2 14^ Given that these risk scores incorporate known cardiovascular risk factors and were primarily developed to predict the risk of major adverse cardiovascular outcomes, it is perhaps not surprising that they also improve the identification of patients with coronary artery disease in the emergency department. Nevertheless, no single risk score we evaluated had optimal rule-in and rule-out performance for obstructive coronary artery disease, and implementing multiple risk scores for this purpose would be challenging in practice. Developing novel risk stratification tools specifically for this group of patients could overcome this current limitation of existing risk scores.

Despite varied derivation populations, outcome measures and timeframes, no single approach to risk stratification clearly outperformed others. This may be due to overlap in the utilisation of known major risk factors for atherosclerosis between scores, limitations inherent in generalising from any single derivation cohort to a wider population and the aetiological role of coronary disease in both short-term and long-term cardiovascular outcomes.

We acknowledge that there are limitations to our analysis. Our cohort of 167 patients is smaller than many of the studies in which these risk scores were derived, and we therefore opted not to derive new cut-points optimised for discrimination of coronary artery disease, although there was no clear inflection point on ROC analysis of any risk score. A population-specific tool should be developed using an appropriately sized study to ensure robust and generalisable findings. We did not have high-sensitivity cardiac troponin T measurements for this cohort and so could not include an analysis of the Troponin-only Manchester Acute Coronary Syndromes score.^36^ The EDACS and TIMI scores use a previous history of varying degrees of coronary artery disease as components of the score, which may inflate their performance. However, in a sensitivity analysis restricted to patients without previously known coronary artery disease, the NPV and PPV for these scores remained similar. While the risk scores evaluated here were designed to predict the risk of short-term or long-term clinical outcomes rather than to diagnose coronary artery disease, this is the underlying pathophysiological basis of the majority of adverse cardiovascular events, and the diagnosis of coronary artery disease is important to patients with chest pain and can facilitate the targeting of preventative therapies that could reduce the risk of these outcomes.

## Conclusions

In patients with intermediate cardiac troponin concentrations in whom myocardial infarction has been ruled out, risk scores can help identify patients with and without obstructive coronary artery disease, but the predictive values of established risk thresholds are not clinically useful. Effective discrimination of patients in this group may require the development of a bespoke approach to risk stratification.

## supplementary material

10.1136/openhrt-2024-002755 online supplemental file 1

## Data Availability

Data are available upon reasonable request.
