# Supplementary material for: Risk scores and coronary artery disease in patients with suspected acute coronary syndrome and intermediate cardiac troponin concentrations
Source: Open Heart. 2024 Aug 1;11(2):e002755. doi: 10.1136/openhrt-2024-002755 (PMC11298728; doi:10.1136/openhrt-2024-002755)
Supplement: online supplemental file 1 [file openhrt-11-2-s001.pdf]

SUPPLEMENTAL MATERIAL

Risk scores and coronary artery disease in patients with suspected acute coronary syndrome and intermediate cardiac troponin concentrations

Daniel Perez-Vicencio, MSc,[1,2*] Alexander JF Thurston, BMBCh,[3*]
Dimitrios Doudesis, PhD,[1,2] Rachel O'Brien, BN,[4] Amy V. Ferry, PhD,[1]
Takeshi Fujisawa, PhD,[1] Michelle C. Williams, MBCHB,[1,5] Alasdair J. Gray, MD,[2,4] Nicholas L Mills, MD,[1,2] Kuan Ken Lee, MD[1,3]

[1] BHF/University Centre for Cardiovascular Science, University of Edinburgh, Edinburgh, UK
[2] Usher Institute, University of Edinburgh, Edinburgh, UK
[3] Edinburgh Heart Centre, Royal Infirmary of Edinburgh, Edinburgh, UK
[4] Department of Emergency Medicine, Emergency Medicine Research Group, Royal Infirmary of Edinburgh, Edinburgh, UK
[5] Edinburgh Imaging Facility, University of Edinburgh, Edinburgh, UK

[*]Contributed equally

Corresponding Author:
Dr Kuan Ken Lee
BHF/University Centre for Cardiovascular Science
The University of Edinburgh
Edinburgh EH16 4SA
United Kingdom
Telephone: 0044 131 242 6515
E-mail: ken.lee@ed.ac.uk

Supplemental Texts: 1

Supplemental Tables: 6

Supplemental Figures: 4

Supplemental Text 1. Risk scores calculation

To our knowledge, each risk score was calculated using the most recent version reported in the literature [1-7]. An R function was created for each risk score to allow for row wise calculation of risk scores in all patients. The R package "RiskScorescvd" was developed for the reproducibility of this analysis using any adequate dataset (https://github.com/dvicencio/RiskScorescvd)[8]. After calculating each patient's risk score, risk classification was assigned based on the corresponding thresholds. According to the ACC guidelines 2023, a HEART score of ≤3 or EDACS <16 discharge without further testing is reasonable, provided access to rapid follow-up is available [9]. We therefore stratified the classifications to binary, either "lowest risk" (HEART ≤3, EDACS <16 [further steps are recommended for EDACS, ***Table A***], GRACE ≤88, ASCVD <.05 or 5%, SCORE2 <5%, SCORE2-OP<7.5% [***Table B***]) or "above lowest risk". An exploration of the data was performed to ensure all variables required to calculate the risk scores were available or could be derived (***Figure 1 and Supplemental Figure 2)***. When derivation was required, insight from two clinicians was given. Missing data values were imputed using MICE R package following the protocol suggested by Jakobsen et. al [10] (***Figure 2B***).

Table A. EDACS additional considerations to categorise patients**.**

Low risk cohort:	Not low risk cohort:
EDACS <16 and	EDACS ≥16 or
ECG shows no new ischaemia and	ECG shows new ischaemia or
0-hr and 2-hr troponin both negative.	0-hr or 2-hr troponin positive.

Table B. SCORE2/OP additional considerations to categorise patients in the UK based on age.

	Low risk	Moderate Risk	High risk
**< 50 years**	<2.5%	2.5 to <7.5%	≥7.5%
**50 – 69 years**	<5%	5 to <10%	≥10%
**≥ 70 years**	<7.5%	7.5 to <15%	≥15%

Supplemental Table 1. Baseline characteristics patients without known previous coronary artery disease stratified by findings on coronary computed tomography angiography.

**Variable**	**All participants,** (n = 103)	**No coronary artery disease,** (n = 42)	**Any coronary artery disease**, (n = 61)	**p-value**[1]
**Female**	**35 (34%)**	**17 (40%)**	**18 (30%)**	**0.25**
**Age**	**61 (13)**	**55 (12)**	**65 (11)**	**<0.001**
**Presenting symptom**				
Chest Pain	84 (82%)	32 (76%)	52 (85%)	0.24
Anginal symptoms	42 (41%)	19 (45%)	23 (38%)	0.44
**Cardiovascular risk factor**				
BMI kg/m2	29.5 (5.9)	29.9 (4.9)	29.2 (6.5)	0.33
Current or previous cigarrete smoker	52 (50%)	16 (38%)	36 (59%)	0.037
Hypertension	41 (40%)	10 (24%)	31 (51%)	0.006
Diabetes	12 (12%)	3 (7.1%)	9 (15%)	0.35
Hyperlipidemia	27 (26%)	10 (24%)	17 (28%)	0.70
Family history	33 (32%)	16 (38%)	17 (28%)	0.27
Chronic kidney disease	7 (6.8%)	2 (4.8%)	5 (8.2%)	0.70
**Medical history**				
Angina	0 (0%)	0 (0%)	0 (0%)	
Myocardial infarction	0 (0%)	0 (0%)	0 (0%)	
Stroke	5 (4.9%)	2 (4.8%)	3 (4.9%)	>0.99
Peripheral vascular disease	1 (1.0%)	0 (0%)	1 (1.6%)	>0.99
**Physiology and investigations**				
Myocardial ischemia on ECG	6 (5.8%)	1 (2.4%)	5 (8.2%)	0.40
T-wave inversion	11 (11%)	3 (7.5%)	8 (13%)	0.52
Heart rate, bmp	80 (18)	78 (20)	81 (16)	0.36
Systolic BP, mm Hg	153 (26)	153 (21)	152 (29)	0.42
Hemoglobin, g/L	143 (14)	143 (16)	143 (12)	0.73
Creatine	78 (16)	80 (19)	77 (14)	0.63
eGFR, mL/min/1.73 m2	85 (17)	87 (19)	84 (16)	0.43
Total cholesterol, mmol/L	5.17 (1.13)	5.29 (0.89)	5.09 (1.27)	0.30
LDL cholesterol, mmol/L	3.25 (1.05)	3.41 (0.90)	3.14 (1.13)	0.27
Peak troponin I concentration, ng/L	8 (6, 12)	8 (7, 13)	8 (6, 11)	0.63
**Clinical risk scores**				
HEART	4 (3, 4)	3 (2, 4)	4 (3, 5)	0.001
EDACS	14 (12, 20)	13 (9, 17)	17 (12, 21)	0.011
GRACE	72 (56, 89)	57 (44, 72)	85 (67, 100)	<0.001

Supplemental Table 1. Baseline characteristics patients without known previous coronary artery disease stratified by findings on coronary computed tomography angiography.

**Variable**	**All participants,** (n = 103)	**No coronary artery disease,** (n = 42)	**Any coronary artery disease**, (n = 61)	**p-value**[1]
TIMI	1 (0, 1)	0 (0, 1)	1 (0, 1)	<0.001
PCE	0.15 (0.07, 0.32)	0.08 (0.04, 0.16)	0.22 (0.11, 0.34)	<0.001
SCORE2/OP	8 (5, 13)	5 (4, 9)	12 (6, 17)	<0.001

Values are median (interquartile range), n (%) or mean ± SD. [1] Pearson's Chi-squared test; Wilcoxon rank sum test; Fisher's exact test. BMI = body mass index; BP = blood pressure; bmp = beats per minute; CABG = coronary artery bypass grafting; CAD = coronary artery disease; ECG = electrocardiogram; EDACS = Emergency Department Assessment of Chest Pain Score; eGFR = estimated glomerular filtration rate; GRACE 2.0 = Global Registry of Acute Coronary Events version 2.0; HEART = History, ECG, Age, Risk factors, Troponin; LDL = low-density lipoprotein; PCE = pooled cohort equations; PCI = percutaneous coronary intervention; SCORE2/OP = Systematic COronary Risk Evaluation 2/Older Population; TIMI = Thrombolysis In Myocardial Infarction.

Supplemental Table 2. Baseline characteristics of patients stratified by the presence or absence of obstructive coronary artery disease.

Variable	All participants, (n = 167)	No coronary artery disease, (n = 47)	Non-obstructive coronary artery disease, (n = 70)	Obstructive coronary artery disease, (n = 50)	p-value[1]
**Female**	46 (28%)	19 (40%)	18 (26%)	9 (18%)	0.044
**Age**	64 (12)	56 (13)	65 (11)	70 (10)	<0.001
**Presenting symptom**					
Chest Pain	143 (86%)	37 (79%)	60 (86%)	46 (92%)	0.19
Anginal symptoms	76 (46%)	21 (45%)	32 (46%)	23 (46%)	>0.99
**Cardiovascular risk factor**					
BMI kg/m2	29.3 (5.8)	29.6 (5.2)	29.7 (6.3)	28.5 (5.6)	0.51
Current or previous cigarrete smoker	89 (53%)	18 (38%)	38 (54%)	33 (66%)	0.026
Hypertension	79 (47%)	11 (23%)	39 (56%)	29 (58%)	<0.001
Diabetes	30 (18%)	3 (6.4%)	14 (20%)	13 (26%)	0.027
Hyperlipidaemia	33 (20%)	10 (22%)	17 (24%)	6 (12%)	0.22
Family history	64 (38%)	17 (36%)	29 (41%)	18 (36%)	0.83
Chronic kidney disease	19 (11%)	2 (4.3%)	8 (11%)	9 (18%)	0.10
**Medical history**					
Angina	34 (20%)	2 (4.3%)	14 (20%)	18 (36%)	<0.001
Myocardial infarction	40 (24%)	3 (6.4%)	16 (23%)	21 (42%)	<0.001
Stroke/TIA	13 (7.8%)	2 (4.3%)	6 (8.6%)	5 (10%)	0.58
Peripheral vascular disease	6 (3.6%)	0 (0%)	1 (1.4%)	5 (10%)	0.021
**Previous revascularization**					
PCI	40 (24%)	3 (6.4%)	15 (21%)	22 (44%)	<0.001
CABG	10 (6.0%)	0 (0%)	7 (10%)	3 (6.0%)	0.071
**Physiology and investigations**					
Myocardial ischaemia on ECG	8 (4.8%)	3 (6.4%)	4 (5.7%)	1 (2.0%)	0.60
ST depression	0 (0%)	0 (0%)	0 (0%)	0 (0%)	
T-wave inversion	24 (15%)	5 (11%)	8 (12%)	11 (22%)	0.24
Heart rate, bmp	76 (18)	77 (20)	78 (17)	73 (18)	0.49
Systolic BP, mm Hg	151 (27)	152 (22)	152 (31)	150 (26)	0.95
Haemoglobin, g/L	143 (15)	143 (16)	144 (14)	143 (14)	0.94
Creatine	82 (19)	79 (19)	81 (16)	86 (21)	0.21
eGFR, mL/min/1.73 m2	82 (18)	87 (18)	82 (17)	77 (16)	0.018
Total cholesterol, mmol/L	4.82 (1.18)	5.18 (0.95)	4.80 (1.27)	4.50 (1.17)	0.016
LDL cholesterol, mmol/L	2.96 (1.18)	3.30 (0.94)	2.97 (1.17)	2.66 (1.32)	0.028

Supplemental Table 2. Baseline characteristics of patients stratified by the presence or absence of obstructive coronary artery disease.

**Variable**	**All participants,** (n = 167)	**No coronary artery disease,** (n = 47)	**Non-obstructive coronary artery disease,** (n = 70)	**Obstructive coronary artery disease,** (n = 50)	**p-value**[1]
Peak troponin I concentration, ng/L	8 (6, 12)	8 (7, 13)	8 (6, 12)	8 (7, 12)	0.92
**Clinical risk scores**					
HEART	4 (3, 5)	3 (2, 4.50)	4 (4, 5)	5 (4, 5)	<0.001
EDACS	17 (12, 21)	14 (10.8, 17)	17 (12, 21)	18 (14, 24)	0.002
GRACE	78 (64, 96)	62 (45, 73)	84 (67, 98)	88 (77, 108)	<0.001
TIMI	1 (0, 2)	0 (0, 1)	1 (1, 2)	2 (1, 3)	<0.001
PCE	0.19 (0.08, 0.36)	0.09 (0.04, 0.16)	0.20 (0.11, 0.36)	0.32 (0.16, 0.45)	<0.001
SCORE2/OP	10.5 (5.0, 16.0)	5.0 (4.0, 9.0)	11.5 (6.0, 17.0)	15.0 (8.5, 18.8)	<0.001

Values are median (interquartile range), n (%) or mean ± SD. [1] One-way ANOVA; Wilcoxon rank sum test; Fisher's exact test. BMI = body mass index; BP = blood pressure; bmp = beats per minute; CABG = coronary artery bypass grafting; CAD = coronary artery disease; ECG = electrocardiogram; EDACS = Emergency Department Assessment of Chest Pain Score; eGFR = estimated glomerular filtration rate; GRACE 2.0 = Global Registry of Acute Coronary Events version 2.0; HEART = History, ECG, Age, Risk factors, Troponin; LDL = low-density lipoprotein; PCE = pooled cohort equations; PCI = percutaneous coronary intervention; SCORE2/OP = Systematic COronary Risk Evaluation 2/Older Population; TIMI = Thrombolysis In Myocardial Infarction.

Supplemental Table 3. Diagnostic performance of risk scores lowest thresholds for obstructive coronary artery disease

Risk Scores (Threshold)	True Positive	False Positive	True Negative	False Negative	Negative Predictive Value (95% CI)	Positive Predictive Value (95% CI)	Sensitivity (95% CI)	Specificity (95% CI)	Proportion Ruled Out	AUC
HEART (<4)	47	73	44	3	93.6 (85.7 - 100)	39.4 (31.1 - 48.2)	94.0 (86.7 - 100)	37.7 (28.5 - 47.7)	28%	0.695 (0.618 - 0.772)
EDACS (<16)*	37	66	51	13	79.4 (68.4 - 89.1)	36.1 (27.5 - 45.3)	73.7 (61.0 - 85.7)	43.8 (34.5 - 52.6)	38%	0.649 (0.555 - 0.743)
GRACE 2.0 (≤88)	27	38	79	23	77.3 (69.1 - 85.1)	41.9 (30.4 - 54.2)	53.9 (39.5 - 67.5)	67.8 (59.5 - 76.1)	61%	0.693 (0.608 - 0.778)
TIMI (=0)	48	76	41	2	95.2 (87.2 - 100)	38.9 (30.6 - 47.5)	95.9 (90 - 100)	35.1 (26.3 - 44.6)	26%	0.730 (0.653 - 0.808)
PCE (<5%)	49	100	17	1	94.1 (81.2 - 100)	33.1 (26.1 - 40.4)	97.9 (93.5 - 100)	14.5 (8.5 - 21.6)	11%	0.714 (0.631 - 0.797)
SCORE2/OP (<5%)*	47	97	20	3	86.7 (71.4 - 100)	32.8 (25.2 - 40.6)	93.9 (86.8 - 100)	17.1 (10.7 - 24.8)	14%	0.714 (0.631 - 0.796)

Values are (Rule out threshold); ASCVD = Atherosclerosis Cardiovascular Disease; AUC = area under the curve; CI = confidence interval; EDACS = Emergency Department Assessment of Chest Pain Score; GRACE 2.0 = Global Registry of Acute Coronary Events version 2.0; HEART = History, ECG, Age, Risk factors, Troponin; SCORE2/OP = Systematic COronary Risk Evaluation 2/Older Population; TIMI = Thrombolysis In Myocardial Infarction. *When EDACS and SCORE2/OP applied further criteria are recommended (Supplement Tables 1 and 2).

Supplemental Table 4. Diagnostic performance of risk scores lowest thresholds for any coronary artery disease

Risk Scores (Threshold)	True Positive	False Positive	True Negative	False Negative	Negative Predictive Value (95% CI)	Positive Predictive Value (95% CI)	Sensitivity (95% CI)	Specificity (95% CI)	Proportion Ruled Out	AUC
HEART (<4)	100	20	27	20	57.3 (43.2 - 71.4)	83.4 (77.1 - 89.8)	83.3 (76.5 - 89.7)	57.5 (43.7 - 72.2)	28%	0.742 (0.655 - 0.829)
EDACS (<16)*	82	21	26	38	40.3 (27.3 - 52.8)	79.7 (72.0 - 87.0)	68.1 (59.8 - 76.2)	55.4 (39.5 - 69.1)	38%	0.684 (0.597 - 0.772)
GRACE 2.0 (≤88)	55	10	37	65	36.2 (26.9 - 45.9)	84.9 (75.5 - 93.0)	45.7 (36.6 - 54.4)	79.1 (66.7 - 90.2)	61%	0.714 (0.624 - 0.804)
TIMI (=0)	104	20	27	16	62.4 (48.8 - 77.3)	84.0 (77.7 - 90.4)	86.6 (80.6 - 92.0)	57.5 (42.2 - 72.7)	26%	0.784 (0.713 - 0.854)
PCE (<5%)	114	35	12	6	66.4 (44.4 - 87.5)	76.6 (69.5 - 83.3)	94.9 (90.7 - 98.4)	25.6 (13.2 - 38.6)	11%	0.754 (0.675 - 0.833)
SCORE2/OP (<5%)*	108	36	11	12	47.5 (26.3 - 68.2)	75.1 (67.9 - 81.9)	89.9 (84.1 - 95.1)	23.4 (11.1 - 36.2)	14%	0.757 (0.680 - 0.834)

Values are (Rule out threshold); ASCVD = Atherosclerosis Cardiovascular Disease; AUC = area under the curve; CI = confidence interval; EDACS = Emergency Department Assessment of Chest Pain Score; GRACE 2.0 = Global Registry of Acute Coronary Events version 2.0; HEART = History, ECG, Age, Risk factors, Troponin; SCORE2/OP = Systematic COronary Risk Evaluation 2/Older Population; TIMI = Thrombolysis In Myocardial Infarction. *When EDACS and SCORE2/OP applied further criteria are recommended (Supplement Tables 1 and 2).

Supplemental Table 5. Diagnostic performance of risk scores lowest thresholds for any coronary artery disease in intermediate-risk patients without known previous coronary artery disease

Risk Scores (Threshold)	True Positive	False Positive	True Negative	False Negative	Negative Predictive Value (95% CI)	Positive Predictive Value (95% CI)	Sensitivity (95% CI)	Specificity (95% CI)	Proportion Ruled Out	AUC
HEART (<4)	41	15	27	20	57.2 (43.1 - 70.7)	73.0 (61.1 - 83.6)	67.0 (55.4 - 77.8)	64.1 (50.0 - 78.4)	46%	0.701 (0.599 - 0.803)
EDACS (<16)*	38	17	25	23	51.9 (37.8 - 66.0)	68.9 (56.6 - 80.8)	62.3 (50.8 - 73.8)	59.0 (44.4 - 73.3)	47%	0.658 (0.551 - 0.764)
GRACE 2.0 (≤88)	25	6	36	36	50.0 (38.0 - 61.1)	80.7 (65.4 - 93.3)	41.1 (28.8 - 53.3)	85.7 (73.5 - 95.5)	70%	0.712 (0.611 - 0.814)
TIMI (=0)	45	15	27	16	62.7 (47.8 - 76.6)	75.0 (63.6 - 85.5)	73.6 (62.5 - 84.7)	64.3 (50.0 - 79.0)	42%	0.708 (0.615 - 0.801)
PCE (<5%)	58	31	11	3	79.2 (55.5 - 100.0)	65.1 (55.5 - 74.7)	95.3 (89.3 - 100)	25.9 (13.3 - 40.0)	14%	0.742 (0.645 - 0.838)
SCORE2/OP (<5%)*	55	32	10	6	62.6 (38.1 - 85.7)	63.3 (53.3 - 73.2)	90.2 (82.4 - 96.8)	23.9 (11.4 - 37.5)	16%	0.753 (0.659 - 0.846)

Values are (Rule out threshold); ASCVD = Atherosclerosis Cardiovascular Disease; AUC = area under the curve; CI = confidence interval; EDACS = Emergency Department Assessment of Chest Pain Score; GRACE 2.0 = Global Registry of Acute Coronary Events version 2.0; HEART = History, ECG, Age, Risk factors, Troponin; SCORE2/OP = Systematic COronary Risk Evaluation 2/Older Population; TIMI = Thrombolysis In Myocardial Infarction. *When EDACS and SCORE2/OP applied further criteria are recommended (Supplement Tables 1 and 2).

Supplemental Table 6. Diagnostic performance of risk scores lowest thresholds for obstructive coronary artery disease in intermediate-risk patients without known previous coronary artery disease

Risk Scores (threshold)	True Positive	False Positive	True Negative	False Negative	Negative Predictive Value (95% CI)	Positive Predictive Value (95% CI)	Sensitivity (95% CI)	Specificity (95% CI)	Proportion Ruled Out	AUC
HEART (<4)	15	41	44	3	93.6 (85.7 - 100.0)	26.7 (15.7 - 39.3)	83.1 (62.5 - 100)	51.8 (41.7 - 62.7)	46%	0.647 (0.543 - 0.752)
EDACS (<16)*	12	43	42	6	87.5 (78.0 - 95.8)	21.7 (11.3 - 33.3)	66.6 (42.8 - 88.0)	49.2 (38.8 - 59.6)	47%	0.641 (0.488 - 0.795)
GRACE 2.0 (≤88)	9	22	63	9	87.5 (79.4 - 94.3)	29.0 (13.3 - 46.0)	49.9 (26.3 - 73.3)	74.0 (63.5 - 82.8)	70%	0.727 (0.604 - 0.851)
TIMI (=0)	16	44	41	2	95.5 (88.6 - 100.0)	26.8 (16.1 - 37.9)	89.2 (72.7 - 100.0)	48.4 (37.6 - 59.3)	42%	0.671 (0.565 - 0.778)
PCE (<5%)	18	71	14	0	100.0 (100.0 - 100.0)	20.1 (12.2 - 28.6)	100.0 (100 - 100)	16.2 (8.5 - 24.4)	14%	0.747 (0.639 - 0.856)
SCORE2/OP (<5%)*	18	69	16	0	100.0 (100.0 - 100.0)	20.7 (12.5 - 29.5)	100.0 (100 - 100)	18.9 (11.1 - 27.7)	16%	0.746 (0.639 - 0.853)

Values are (Rule out threshold); ASCVD = Atherosclerosis Cardiovascular Disease; AUC = area under the curve; CI = confidence interval; EDACS = Emergency Department Assessment of Chest Pain Score; GRACE 2.0 = Global Registry of Acute Coronary Events version 2.0; HEART = History, ECG, Age, Risk factors, Troponin; SCORE2/OP = Systematic COronary Risk Evaluation 2/Older Population; TIMI = Thrombolysis In Myocardial Infarction. *When EDACS and SCORE2/OP applied further criteria are recommended (Supplement Tables 1 and 2).

Supplemental Figure 1. Summary of commonly used clinical risk scores and their calculations. *ARCHITECTSTAT troponin I assay; Abbott Laboratories. † The risk factors only apply to patients 18 – 50. **Undergoes equation transformation based on age. C represents a constant; BP blood pressure; yo years old; TnI Troponin I; chol cholesterol; HDL High Density Lipoprotein; ECG Electrocardiogram; HEART, History, ECG, Age, Risk factors, Troponin; EDACS, Emergency Department Assessment of Chest Pain Score; TIMI Thrombolysis In Myocardial Infarction; GRACE 2.0 Global Registry of Acute Coronary Events version 2.0; ASCVD Atherosclerosis Cardiovascular Disease; SCORE2/OP Systematic COronary Risk Evaluation 2/Older Population.

	HEART	EDACS	TIMI	GRACE 2.0	PCE	SCORE2/OP
Troponin*/ biomarker	TnI<br>≤ normal limit: 0<br>1 - 3x normal limit: 1<br>>3x normal limit: 2	TnI 0hr or 2hr > 99th %: High Risk	TnI > 99th %: 1	TnI > 99th %: 13<br>Creatine x C: 0–28	Log(Total chol) x C^{tc}<br>Log(HDL) x C^{H}	Total chol x C^{tc**}<br>HDL x C^{H**}
Risk/ History	No typical features: 0<br>Mixed typical/non - typical: 1<br>Mostly typical features: 2	†Coronary disease or risk factors ≥3: 4<br>Diaphoresis: 3	Risk factors ≥3: 1<br>Aspirin use: 1<br>Known CAD (stenosis ≥50 %): 1	Killip class: 0–44<br>Cardiac arrest: 30	Diabetes: 1 x C^{d}<br>Smoker: 1 x C^{s}<br>Coefficients (C^{x}) change depending on Race: Black or White	Diabetes: 1 x C^{d}<br>Smoker: 1 x C^{s}
Age	< 45 yo: 0<br>45 to 64 yo: 1<br>≥65 yo: 2	18 to 45 yo: 2<br>46 to 85 yo: 4–18<br>≥86 yo: 20	<65 yo: 0<br>65 to 74 yo: 2<br>≥75 yo: 3	<90 yo x C: 0–98<br>≥90 yo: 100	Log(Age) x C^{a}<br>Log(Age) x Log(all)$^{\$}$	Coefficients (C^{x}) change depending of Age: <70 or ≥70
Sex	-	Female: 0<br>Male: 6	-	-	Coefficients (C^{x}) change depending on sex: Female or Male	Coefficients (C^{x}) change depending of sex: Female or Male
Physiology	Normal ECG: 0<br>Abnormal ECG: 1<br>ST depression: 2	Typical pain radiation: 5<br>Worse pain on inspiration: - 4<br>Pain by palpation: - 6	Severe angina: 1<br>ECG ST Elevation or LBBB: 1	Heart rate x C: 0–34<br>Systolic BP x C: 0–40<br>ST change: 17	Log(Systolic BP) x C^{bpw} or C^{bpwo} depending on treatment	Systolic BP x C^{bp**}
Low risk Criteria	**Sum**<br>< 4	**Sum**<br>< 16<br>Negative 0 & 2h TnI	**Sum**<br>= 0	**Equation (recalibration)**<br>< 89	**Equation (recalibration)**<br>< 5%	**Equation (recalibration)**<br>< 2.5% for ages < 70<br>< 7.5% for ages ≥ 70

Supplemental Figure 2. Required variables allocation and missing frequencies. A) Heat map of variables used for each risk score calculation. Required variables shown in blue. B) Frequency of missing values

A) **B)**

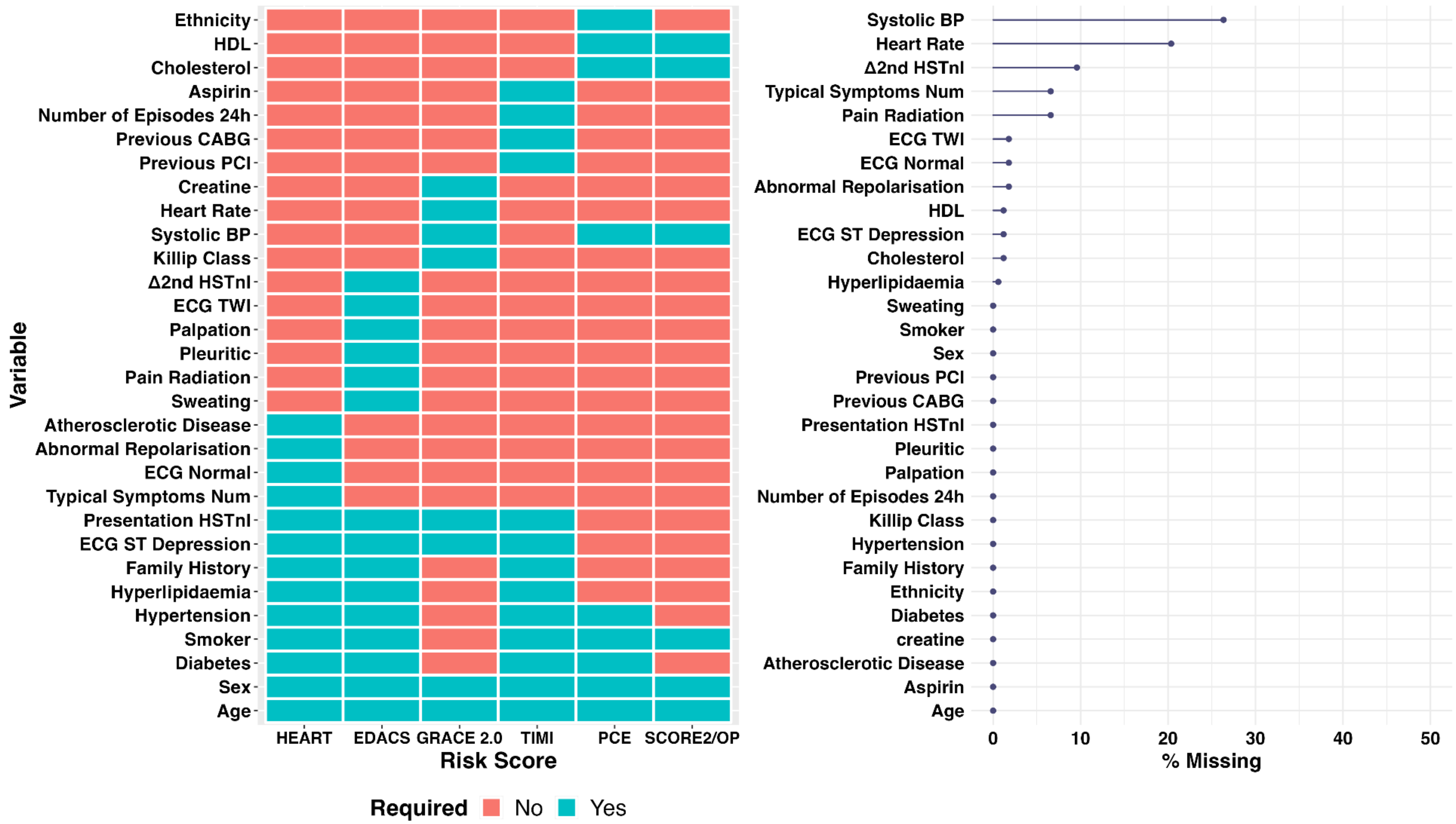

Supplemental Figure 3. Comparison of AUC performances using DeLong's Test. The heat map provides a visual representation of the statistical differences between the ROC curves for risk scores for A) any coronary artery disease and B) obstructive coronary artery disease. AUC = area under the curve.

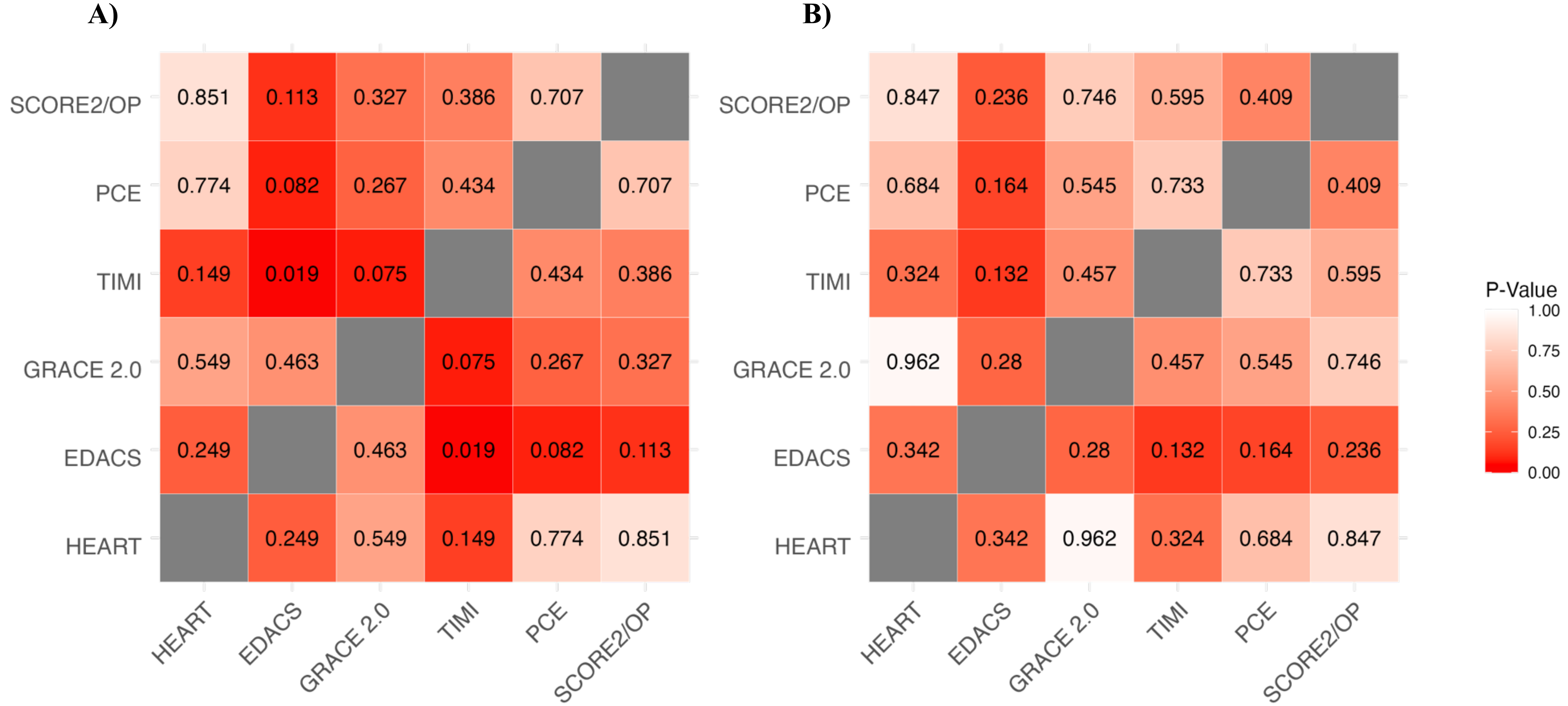

Supplemental Figure 4. ROC analysis of risk scores for an adjudicated diagnosis of any coronary artery disease and obstructive coronary artery disease restricted to intermediate-risk patients without known previous coronary artery disease. Receiver-operating-characteristic (ROC) curves illustrating discrimination of troponin and risk scores for A) any coronary artery disease and B) obstructive coronary artery disease. AUC = area under the curve.

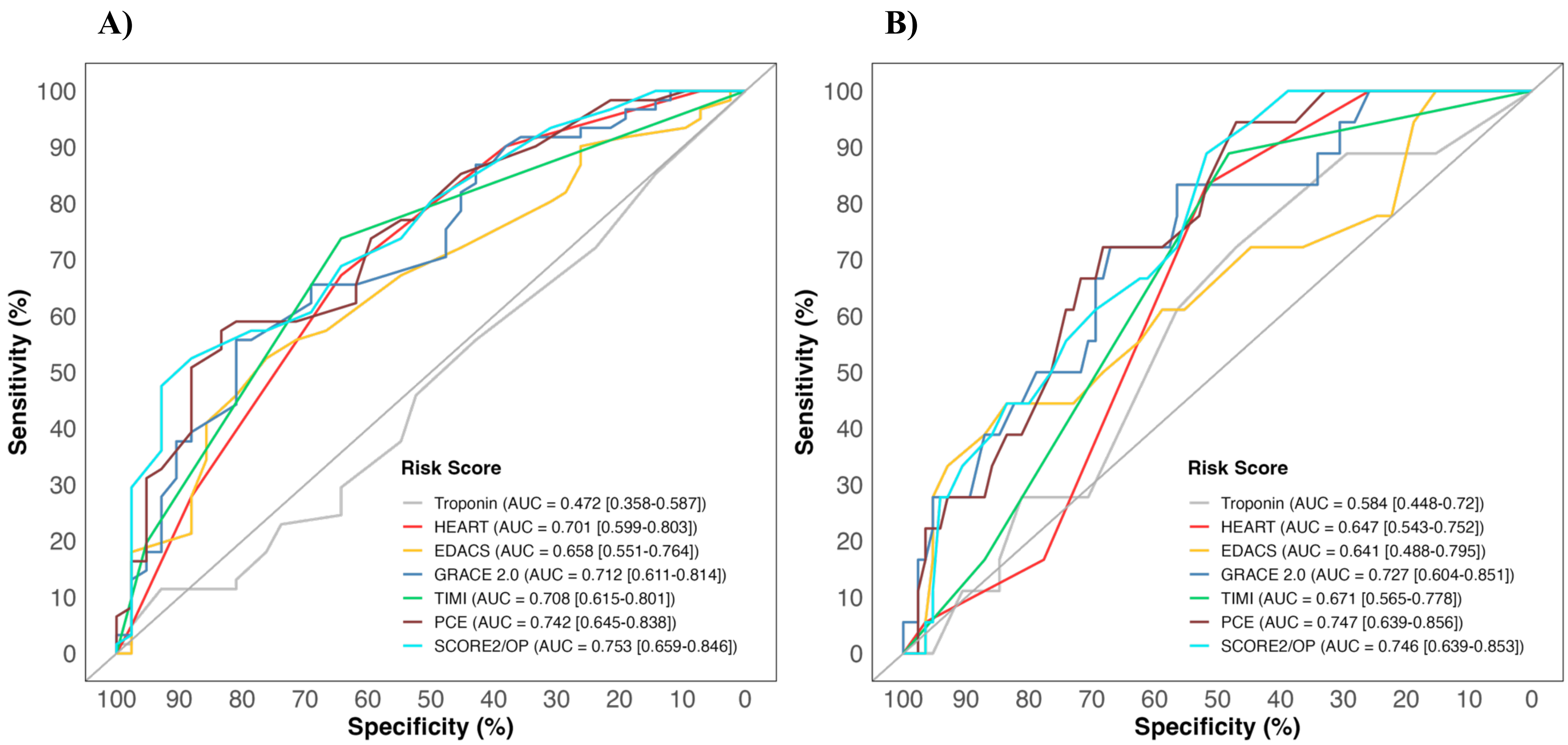

References

1. Than, M., et al., *Development and validation of the Emergency Department Assessment of Chest pain Score and 2 h accelerated diagnostic protocol.* Emergency Medicine Australasia, 2014. **26**(1): p. 34-44.
2. Hageman, S., et al., *SCORE2 risk prediction algorithms: new models to estimate 10-year risk of cardiovascular disease in Europe.* European Heart Journal, 2021. **42**(25): p. 2439-2454.
3. Six, A.J., B.E. Backus, and J.C. Kelder, *Chest pain in the emergency room: value of the HEART score.* Neth Heart J, 2008. **16**(6): p. 191-6.
4. Karmali, K.N., et al., *A systematic examination of the 2013 ACC/AHA pooled cohort risk assessment tool for atherosclerotic cardiovascular disease.* J Am Coll Cardiol, 2014. **64**(10): p. 959-68.
5. Fox, K.A.A., et al., *Should patients with acute coronary disease be stratified for management according to their risk? Derivation, external validation and outcomes using the updated GRACE risk score.* Bmj Open, 2014. **4**(2).
6. de Vries, T.I., et al., *SCORE2-OP risk prediction algorithms: estimating incident cardiovascular event risk in older persons in four geographical risk regions.* European Heart Journal, 2021. **42**(25): p. 2455-2467.
7. Antman, E.M., et al., *The TIMI risk score for unstable angina/non-ST elevation MI: A method for prognostication and therapeutic decision making.* JAMA, 2000. **284**(7): p. 835-42.
8. Daniel Perez-Vicencio, D.D.a.A.J.T., *RiskScorescvd: Cardiovascular Risk Scores Calculator.* 2023.
9. Writing, C., et al., *2022 ACC Expert Consensus Decision Pathway on the Evaluation and Disposition of Acute Chest Pain in the Emergency Department: A Report of the American College of Cardiology Solution Set Oversight Committee.* J Am Coll Cardiol, 2022. **80**(20): p. 1925-1960.
10. Jakobsen, J.C., et al., *When and how should multiple imputation be used for handling missing data in randomised clinical trials - a practical guide with flowcharts.* Bmc Medical Research Methodology, 2017. **17**.
